# C3 glomerulonephritis associated with monoclonal gammopathy of renal significance: case report

**DOI:** 10.1186/s12882-018-0927-0

**Published:** 2018-06-08

**Authors:** Juana Alonso-Titos, Lara Perea-Ortega, Eugenia Sola, Alvaro Torres-Rueda, Myriam León, Remedios Toledo, Ana D. Duarte, Teresa Vazquez, Maria Dolores Martinez-Esteban, Alicia Bailen, Pedro Ruiz-Esteban, Domingo Hernandez

**Affiliations:** 1 Nephrology Department, Carlos Haya Regional University Hospital, University of Malaga, IBIMA, REDinREN (RD16/0009/0006), Avda. Carlos Haya s/n, E-29010 Malaga, Spain; 2 Pathology Department, Carlos Haya Regional University Hospital, Málaga, Spain; 3 Hematology Department, Carlos Haya Regional University Hospital, Málaga, Spain

**Keywords:** Chronic kidney disease, Monoclonal gammopathy, C3 glomerulonephritis, Alternative complement pathway, Case report

## Abstract

**Background:**

Morbidity associated with monoclonal gammopathy of renal significance is high due to the severe renal lesions and the associated systemic alterations. Accordingly, early diagnosis is fundamental, as is stopping the clonal production of immunoglobulins using specific chemotherapy**.**

**Case presentation:**

A 75-year-old man with chronic renal disease of unknown origin since 2010 experienced rapid worsening of renal function over a period of 6 mos. Bone marrow biopsy showed monoclonal gammopathy of undetermined significance. Kidney biopsy showed the presence of C3 glomerulonephritis, with exclusive deposits of C3 visible on immunofluorescence and a membranoproliferative pattern on light microscopy. Skin biopsy showed endothelial deposition of complement. Given both the renal and cutaneous involvement the patient was considered to have monoclonal gammopathy of renal significance. We considered an underlying pathogenic mechanism for the renal alteration secondary to activation of the alternative complement pathway by the anomalous immunoglobulin. Despite treatment with plasmapheresis, bortezomib and steroids, advanced chronic kidney disease developed.

**Conclusions:**

The possible underlying cause of the monoclonal gammopathy of renal significance suggests that monoclonal gammopathy should be considered in adult patients with membranoproliferative glomerulonephritis.

## Background

Renal alterations, common in paraproteinemias, are characterized by immunoglobulin G (IgG) clonal proliferation generated by B lymphocytes or plasma cells. Multiple kidney disorders can result from the precipitation or deposition of clonal immunoglobulins (usually light-chain), either directly, causing the activation and renal deposition of components of the classical and terminal complement pathway, or indirectly via activation of the components of the complement that are eventually deposited in the kidney [1, 2].

Monoclonal gammopathy of renal significance (MGRS) is a clinico-pathological entity grouping renal alterations secondary to the secretion of a monoclonal immunoglobulin by a B-cell clone but which fails to reach the 10% infiltration necessary to be considered a multiple myeloma. This hematological disorder is generally classified as monoclonal gammopathy of uncertain significance (MGUS). However, this nomenclature has recently been changed to MGRS given the important renal involvement, which can involve primary amyloidosis, membranoproliferative glomerulonephritis due to deposition of monoclonal long chains, or C3 glomerulonephritis (C3-GMN) [1, 3].

Morbidity associated with MGRS is high due to the severe renal lesions and the associated systemic alterations [1, 4]. Accordingly, early diagnosis is fundamental, as is stopping the clonal production of immunoglobulins using specific chemotherapy.

We report a patient with C3-GMN associated with MGRS that gradually evolved to advanced chronic renal failure despite treatment.

## Case presentation

### Clinical history and initial laboratory data

The patient was a 75-year-old man with a history of hypertension, hypertensive cardiopathy, benign prostatic hyperplasia and right renal lithiasis requiring surgical lithotomy. In January 2013, during study for an upper digestive tract hemorrhage, a high-grade gastric gastrointestinal stromal tumor (GIST) was diagnosed, with a spindle-cell pattern, and a duodenal neuroendocrine tumor, requiring total gastrectomy and esophageal-jejunal anastomosis. The extension study showed grade T4, N0, M0. He was treated with imatinib (400 mg/day) continuously for 6 mos. He had chronic kidney failure (serum creatinine 1.7–2.5 mg/dL) since 2010 and IgG kappa paraproteinemia detected in 2013.

In May 2015 he was admitted with rapid worsening of renal function, with serum creatinine of 5 mg/dL (in October 2014 it had been 1.7 mg/dL), proteinuria 524.79 mg/24 h and an IgG kappa monoclonal spike.

The proteinogram detected a monoclonal band in the gamma fraction with a monoclonal spike of 0.56 g/dL and IgG kappa on serum immunoelectrophoresis. Quantification of the free light chains showed kappa chains 962 mg/L, lambda chains 28.8 mg/L, and a free kappa/free lambda ratio of 33.4. Urine immunoelectrophoresis showed 36% monoclonal component, equivalent to 189 mg/24 h, and free kappa light chains.

Serum levels of immunoglobulin A (IgA) and immunoglobulin G (IgG) were within normal ranges, with a slight decrease in immunoglobulin M (IgM) (33 mg/dL). He had marked hypocomplementemia, with reductions in complement C3 (C3) (47 mg/dL) and complement C4 (C4) (22 mg/dL), and slightly raised levels of beta 2 microglobulin (14.4 mg/L). The other parameters were normal or negative.

Bone marrow aspirate showed 1.2% plasma cells with an abnormal phenotype, typical of myelomatous plasma cells, plus 0.2% normal phenotype plasma cells. The bone map showed a marked general reduction in bone density, with non-specific mid-spine vertebral wedging. Flow cytometry discarded monoclonal B-cell lymphoid proliferation.

### Kidney biopsy

**Fig. 1:**
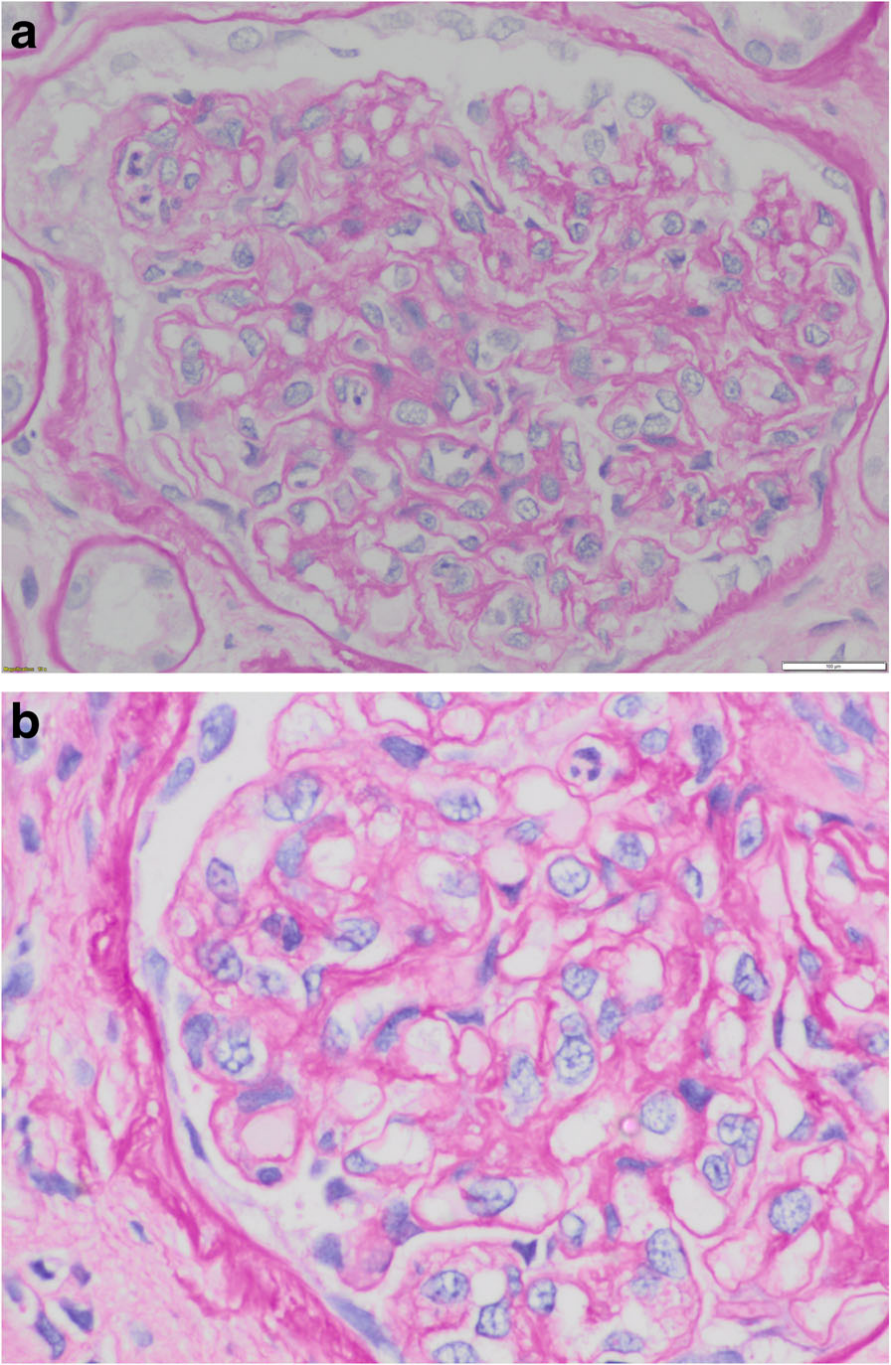
**a** Periodic acid-Schiff: lobular pattern with cell proliferation, a few cases of endocapillary type and others mesangial, with isolated polymorphonuclear neutrophils. Arterioles with focal subendothelial hyalinosis. Marked interstitial fibrosis and tubular atrophy. **b** Periodic acid Schiff staining showed focal double contour (arrow) in most of the glomeruli (× 60)

**Fig. 2:**
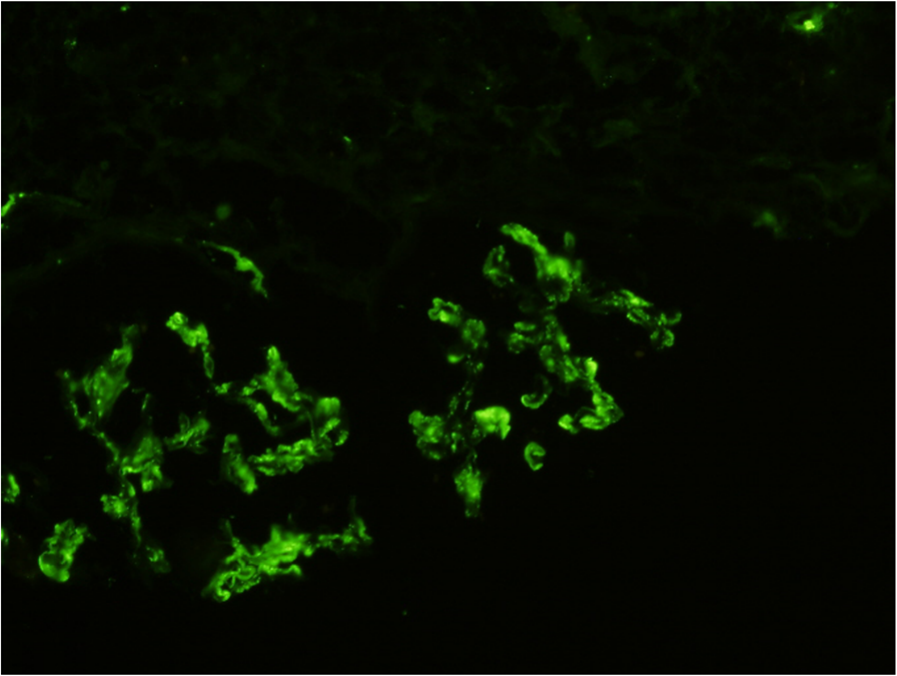
Immunofluorescence showing intense mesangial positivity for C3 (× 40)

Ultrasound-guided percutaneous kidney biopsy 6 days after admission showed alterations compatible with C3-GMN (Figs 1a, b and 2) with no extracapillary proliferation. Under light microscopy the kidney biopsy showed 50% sclerosed glomeruli, with the rest having a lobular pattern with cell proliferation, a few cases with an endocapillary type and others mesangial, with isolated polymorphonuclear neutrophils; arterioles with focal subendothelial hyalinosis; and marked interstitial fibrosis and tubular atrophy (Fig. 1a). Periodic acid-Schiff staining showed focal double contour in most of the glomeruli (Fig. 1b). Immunofluorescence revealed intense mesangial positivity for C3 and negative for IgA, IgG, IgM, fibrinogen and kappa and lambda light chains (Fig. 2). The biopsy sample for electron microscopy study only contained one glomerulus. As this was sclerosed we cannot consider the sample representative.

### Skin biopsy

**Fig. 3:**
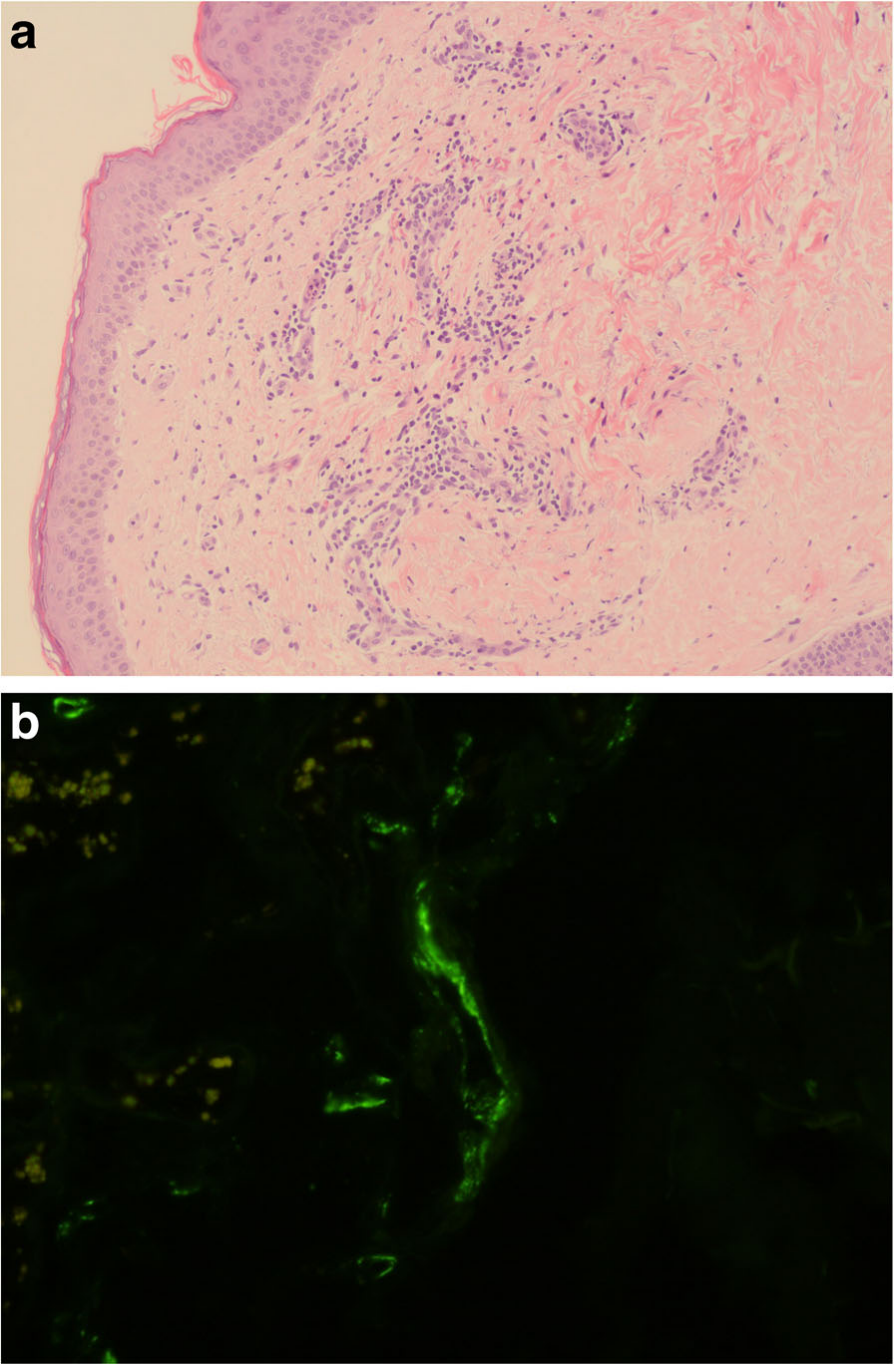
**a** Light microscopy, hematoxylin-eosin stain (H&E × 20): perivascular lymphocyte infiltrate with no evidence of vasculitis. Deposits of C3 in the vessel walls. **b** Immunofluorescence skin: C3 deposits in the vascular walls

As the patient also had purpura-like lesions on the trunk and legs a skin biopsy, taken from the lumbar area, showed a perivascular lymphocyte infiltrate but no evidence of vasculitis, with deposits of C3 in the vessel walls (Fig. 3a and b).

Although the patient had no clear laboratory data for thrombotic microangiopathy (normal haptoglobin and lactate dehydrogenase (LDH)), schistocytes (16 per 1000) and moderate thrombocytopenia were seen on the blood smear.

Given the severity of the clinical and histological picture the patient was treated with high-dose steroids (6-methylprednisolone 500 mg/day for 3 consecutive days) and plasmapheresis (8 complete plasma exchanges). Concomitantly, treatment was started with bortezomib 2 mg/day up to 5 doses, to reduce the production of monoclonal IgG kappa paraprotein and indirectly prevent the activation of the alternative complement pathway. After completing the first chemotherapy cycle with dexamethasone and bortezomib, we noticed a hematological response, with electrophoresis demonstrating disappearance of the monoclonal protein in the serum. The skin lesions have now disappeared. However, despite the treatment the patient is currently on predialysis (serum creatinine 4 mg/dL and glomerular filtration rate 14 mL/min), with proteinuria of 230 mg/24 h, C3 hypocomplementemia (41 mL/dL) and conserved diuresis.

The hospital ethics committee (CEI Provincial de Málaga) is aware of the case, which followed normal clinical practice, and has given its written consent. The patient also gave written informed consent for all the procedures and publication of the case.

## Discussion

Our patient had had chronic kidney disease since 2010, with rapid worsening of renal function in the last 6 mo. Bone marrow aspirate confirmed MGUS. The renal biopsy showed C3-GMN and the skin biopsy revealed endothelial deposition of complement. Indeed, this is a strong point of our study, determining that pathological deposits were produced not only in the kidney but also in the cutaneous blood vessels. These findings are compatible with a paraproteinemic syndrome with no criteria for multiple myeloma. We speculate that the cause is very probably the underlying phenomenon of the activation of the alternative complement pathway, due to the biological activity of the long chain interfering with the regulation of the complement regulatory-inhibitory proteins.

The particularity of this case, as well as its interest, relates to the coexistence of the skin and kidney lesions as a common pathogenic mechanism of dysregulation of the alternative complement pathway, probably produced by the presence of a monoclonal paraprotein. In addition to this, we also show the histological study of both lesions, with the immunofluorescent study of the skin and kidney tissue showing the exclusive deposition of C3. In this regard very few reports exist; we have only found two very recent reviews, though neither provide much histological evidence [5, 6]. The former used immunofluorescence to demonstrate C3 deposits in the skin, though it was only weakly positive. However, in the latter paper, Chauvet et al., who report the evaluation of chemotherapy in a large cohort of patients with C3 glomerulopathy associated with monoclonal gammopathy, mention five cases with additional severe renal symptoms. These were diffuse mucinosis in one patient, digital ischemia in two, purpuric lesions in one and capillary leak syndrome in one. None of these cases had a histological and immunofluorescence study of the skin, which could have demonstrated that the cause of these lesions was activation of the alternative complement pathway and its tissue deposition as a common pathogenic mechanism of the C3 glomerulopathy and the skin at the same time.

The diagnosis of MGUS requires a serum monoclonal paraprotein band < 30 g/L, a bone marrow biopsy showing < 10% abnormal plasma cells, absence of lytic lesions and organ damage, as well as no anemia or hypercalcemia [1, 2, 7]. This syndrome complex is compatible with that of our patient. Given the presence of renal and cutaneous involvement it was considered to be MGRS [8].

The spectrum of kidney lesions associated with monoclonal gammopathy is extensive [1, 4]. The physical and chemical properties of the immunoglobulin produced can result in various glomerular alterations, including membranoproliferative glomerulonephritis, C3-GMN, primary amyloidosis, fibrillary glomerulonephritis (GMN), cryoglobulinemic GMN and disease due to immunoglobulin deposition (light or heavy chains). There may also be indirect renal involvement from the dysregulation of the alternative complement pathway. In this case the monoclonal immunoglobulin inhibits the plasma complement regulatory factors, resulting in deposition of C3 in the renal and systemic capillary vessels, as occurred in our patient. Indeed, this has been suggested as an origin of C3-GMN, a recently described entity, characterized by C3 staining on immunofluorescence and the absence of immunoglobulins in bone marrow, together with a mesangial membranoproliferative and/or endocapillary pattern with electrodense subepithelial, intramembranous and subendothelial deposits [9, 10].

Obviously, these alterations in the regulation of the alternative complement pathway can be secondary to genetic mutations or acquired disorders [10]. A molecular genetic study was undertaken of the complement system. This study consisted of two main tests. The first concerned immunological tests to determine the levels of C3 and C4, complement factor H (CFH), membrane cofactor protein (MCP), complement factor I (CFI) and complement factor B (CFB), as well as a functional analysis of factor H, anti-factor H antibodies, screening for abnormalities and deficiencies in the factor H-related protein family (CFHR). The result of this first part was normal, except for the C3 levels, which were reduced. The second part of this study was a genetic analysis of the complement system, with sequencing of 14 genes: CFH, MCP, CFI, C3, CFB, diacylglycerol kinase E (DGKE), CFHR1, CFHR2, CFHR3, CFHR4, CFHR5, a disintegrin-like and metalloprotease with thrombospondin type 1 (ADAMTS13), thrombomodulin gene (THBD), and complement factor P properdin (CFP). In this genetic study we found no mutations in the complement regulating factors. We did detect a few polymorphisms, including a heterozygotic deletion in the genes CFHR1-CFHR38 (occasionally related with the formation of anti-CFH antibodies), and which, in this case, coincided with the appearance of another polymorphism in intron 12, which encodes for the gene CFH and was 1696 + 2019 G > A in homozygosis, which could indicate the presence of a hybrid gene CFH/CFHR1. This could have been the cause of the dysregulation in the complement system.

Whatever the situation, the pathogenic mechanism by which immunoglobulin can activate the alternative complement pathway is still not completely known. Nor is it known whether the presence of genetic alterations of complement can favor the progression of the kidney lesions in the presence of monoclonal immunoglobulin, as seen in our patient [2].

Zand et al. reported 10 cases diagnosed between 2009 and 2012 at the Mayo Clinic of C3-GMN associated with MGUS. In only two patients did they find circulating C3 nephritic factor (C3NeF). Another two patients had elevated serum levels of soluble membrane-attack complex factors and in six they found functional abnormalities of the alternative complement pathway. In the genetic study, in three patients they identified risk alleles coding for complement factor H (CFH). Thus, the allele Y402H could be a risk polymorphism for this particular entity.

Both Bridox et al. and Zand et al. found that the CFH allele 402H was present in 30% of patients. Although this allele is also present in 60% of the general population, these authors suggest that its presence may represent a risk for C3-GMN in the context of MGRS [2, 11].

The mutation or variation of alleles encoding genes regulating the alternative complement pathway may not be sufficient to trigger the disease, with another triggering factor required, such as an infection or the presence of a circulating monoclonal immunoglobulin. Indeed, this is a limitation of this study as we have not detected the pathogenic mechanism leading to activation of the alternative complement pathway or any genetic mutations. Further studies are therefore needed to elucidate this.

## Conclusion

In conclusion, the relevance of this clinical case is that it describes the different phases of monoclonal gammopathy due to dysregulation of the alternative complement pathway, from renal disorder, with the development of C3-GMN, to systemic endothelial disease with deposition of C3 in blood vessels. The report also demonstrates the importance of establishing early treatment to halt the production of monoclonal immunoglobulin and to prevent the possible serious onset of thrombotic microangiopathy, which we were probably able to prevent by starting treatment at an early stage with plasmapheresis with fresh frozen plasma replacement and corticoid treatment plus bortezomib. The histological confirmation of the disorder is also provided (with skin and renal biopsies). Genetic mutations in the complement factors were ruled out, although there were certain polymorphisms which, in addition to some triggering factor like the production of monoclonal immunoglobulin, could alter the function of the alternative complement pathway.

## Abbreviations

ADAMTS13: A disintegrin-like and metalloprotease with thrombospondin type 1
C3: Complement C3
C3-GMN: C3 glomerulonephritis
C3NeF: C3 nephritic factor
C4: Complement C4
CFB: Complement factor B
CFH: Complement factor H
CFHR: Factor H-related protein family
CFI: Complement factor I
CFP: Complement factor P properdin
DGKE: Diacylglycerol kinase-E
GIST: Gastrointestinal stromal tumor
GMN: Glomerulonephritis
IgA: Immunoglobulin A
IgG: Immunoglobulin G
IgM: Immunoglobulin M
LDH: Lactate dehydrogenase
MCP: Membrane cofactor protein
MGRS: Monoclonal gammopathy of renal significance
MGUS: Monoclonal gammopathy of uncertain significance
THBD: Thrombomodulin gene
